# Transforming spinal surgery: five years of navigation, workflow optimization and clinical impact

**DOI:** 10.3389/fsurg.2026.1761489

**Published:** 2026-04-20

**Authors:** Johannes Groh, Simon Schramm, Lilli Holzmann, Simon Wagner, Mario Perl, Johannes Krause

**Affiliations:** Department of Trauma and Orthopedic Surgery, University Hospital Erlangen, Friedrich-Alexander University Erlangen-Nürnberg (FAU), Erlangen, Germany

**Keywords:** 3D scan, navigation, pedicle screw, spine, trauma

## Abstract

**Background:**

Navigation-assisted spinal instrumentation is increasingly used in modern spine surgery, offering improvements in accuracy, workflow efficiency, and radiation safety. However, real-world implementation and the transition from fluoroscopy to navigation in high-volume trauma centers remain insufficiently described.

**Methods:**

This retrospective single-center study reviewed all dorsal spinal instrumentation procedures performed between 2015 and 2025 at a Level I trauma center. A total of 557 patients were analyzed: 119 navigated and 438 fluoroscopic procedures. Demographics, ASA classification, operative time, screw count, radiation parameters, anatomical distribution, and revision rates were compared, with specific focus on changes after the introduction of navigation in 2020.

**Results:**

Navigation use increased steadily and expanded from lumbar to more anatomically demanding regions. Navigated cases involved older patients with higher ASA scores. Although operative times were longer in navigated procedures, this was explained by higher screw counts, and time per screw did not differ significantly. A clear learning curve was observed, with time per screw improving from 27 (±22) to 19 (±7) minutes (*p* = 0.03). Radiation time was significantly lower in the navigated group, while total dose was comparable. Screw misplacement–related revisions were less frequent with navigation (1% vs. 5%), whereas wound-related revisions were more common, reflecting higher comorbidity and a greater proportion of open procedures.

**Conclusion:**

Navigation substantially altered clinical practice, leading to its predominant use in complex anatomies and higher-risk patients. It improved screw accuracy and reduced radiation exposure while maintaining procedural efficiency after the learning curve. With ongoing advances such as robotics, augmented reality, and markerless registration, the role of navigation in spinal trauma surgery is expected to expand further.

## Introduction

Advances in modern medicine continue to reshape surgical practice, and spine surgery is among the fields most profoundly transformed over the past two decades. The increasing adoption of minimally invasive spine surgery (MISS) has fundamentally changed operative strategies, offering substantial benefits such as reduced approach-related morbidity, decreased blood loss, diminished soft-tissue trauma, and shorter hospital stays for patients (1). Despite these clear clinical advantages, MISS inherently limits the surgeon's visual and tactile orientation, making safe and accurate placement of pedicle screws considerably more demanding. As pedicle screw positioning remains a critical determinant of biomechanical stability and neurological safety, technological solutions that enhance intraoperative orientation have gained substantial importance.

Parallel to the transition toward less invasive surgery, intraoperative imaging has undergone major developments. High-resolution 3-dimensional (3D) imaging systems, including advanced C-arm and cone-beam technologies, have markedly improved the surgeon's ability to control implant position during the procedure (2). Building on these imaging advances, optical navigation has emerged as a key adjunct in spine surgery and is now widely integrated into routine practice. The combination of real-time instrument tracking with volumetric image datasets has demonstrated higher screw placement accuracy and a substantial reduction in radiation exposure for both patients and staff (3, 4). These benefits apply particularly to multilevel constructs, where navigation may also improve workflow efficiency (5).

Reported rates of pedicle screw malposition vary widely depending on pathology and surgical technique, with values ranging from single-digit percentages to over 40% in complex deformity or trauma cases (4, 6, 7). Given that misplacement may lead to neurologic deficits in a notable subset of patients, enhancing the precision of screw insertion remains a central priority.

Image-guided navigation systems provide spatial orientation by linking surgical instruments to pre- or intraoperatively acquired datasets. Modern optical tracking platforms rely on camera-based detection of reflective markers attached to the patient and instruments, enabling continuous three-dimensional visualization with high spatial accuracy (8). Several registration techniques can be used to match the physical operative field to the corresponding dataset. Surface-based registration, commonly applied when preoperative CT data are available, uses characteristic bony landmarks and mathematical algorithms such as the iterative closest point method to achieve alignment (9). Although reliable, this method may prolong operative time and requires sufficient exposure of bone structures, limiting its usefulness in minimally invasive approaches.

The integration of intraoperative 3D imaging with direct navigation linkage resolves many of these limitations. By acquiring the dataset intraoperatively and transferring it automatically to the navigation system, positional discrepancies are minimized and additional exposure for landmark acquisition becomes unnecessary. This workflow is particularly beneficial in percutaneous procedures and trauma settings, where anatomical configurations often change after positioning or reduction (10). Furthermore, modern systems allow fusion of preoperative diagnostic scans with intraoperative datasets, combining detailed planning information with real-time anatomical precision (11).

In recent years, numerous studies have demonstrated the advantages of navigation-assisted screw placement across spinal regions and surgical indications. Reported accuracy rates frequently exceed 95% when navigation is used accompanied by reduced complication rates associated with screw misalignment (4, 11, 12).

Navigation was introduced in our hospital in 2020. Before implementation of the navigation system, all percutaneous spinal procedures in our department were performed under conventional fluoroscopy. Only two surgeons had prior experience with navigated techniques from external institutions, which meant that the introduction of the system represented both a technological and organizational transition. Since 2020, navigation has increasingly been incorporated into daily practice, including urgent and out-of-hours procedures.

Despite the growing body of evidence supporting navigation-assisted spinal instrumentation, the real-world implementation of such systems in trauma-centers is insufficiently characterized.

Therefore, the aim of this study was to analyze the utilization of navigated dorsal spinal instrumentation over a 5-year period at a Level I trauma center and to compare procedural characteristics, efficiency metrics, and radiation parameters with fluoroscopic techniques from the last 10 years. Furthermore, this study assessed how navigation adoption evolved over time, including changes in anatomical indications, surgical complexity, and perioperative logistics.

## Material and methods

As mentioned above, navigation was introduced in our hospital in 2020. The aim was to analyze the usage behavior of dorsal interventions on the spine since the introduction of navigation and to compare it with a 5-year period before the introduction of this system. For this purpose, the IT Department for Research & Management conducted a structured search for all dorsal instrumentation using screw-rod systems on the spine from 2015 to 2025. The OPS codes 5-83b.50 (osteosynthesis of the spine by screw-rod system: 1 segment), 5-83b.51 (2 segments), 5-83b.52 (3 segments), 5-83b.54 (4 segments), 5-83b.55 (5 segments), 5-83b.56 (6 segments), 5-83b.57 (7 to 10 segments) and 5-83b.58 (11 or more segments) were used as search parameters. The patient file was reviewed, and the following parameters were collected:

- Age at surgery
- Sex
- ASA Classification
- Surgery time and duration
- Open/percutaneous
- Fracture height
- Instrumented Height
- Number of screws
- Cement augmentation and additive kyphoplasty
- 3D Scan
- Radiation time and dose
- Revision
- Navigation

The patient names were then pseudonymized, so that no conclusions could be drawn about the identity.

The result was a total collective of *n* = 557 patients.

As spinal surgery at our institution is mostly dedicated to the treatment of traumatic vertebral injuries, the indication in all cases was one or more traumatic spinal fractures requiring surgical stabilization.

Subgroups were divided into “Navigation” (all patients who received a navigated procedure) with a number of *n* = 119 patients, “Fluoroscopic” (all patients who received a non-navigated procedure) with *n* = 438 patients. The latter group was further divided into non-navigated interventions before the introduction of navigation (2015–2019; *n* = 184) and non-navigated interventions after the introduction of navigation (2020 to 2025; *n* = 245).

The choice between fluoroscopic and navigated instrumentation after the implementation was based on surgeon judgment, considering anatomical complexity, spinal region, and fracture morphology. No formal institutional guideline mandated navigation use during the study period.

The statistical analysis was carried out using Microsoft Excel for Office 365 (Microsoft Corp., Redmond, Washington) and IBM SPSS Statistics 25 (IBM Inc., Armonk, NY, USA). Continuous variables were compared using Student's t-test or Mann–Whitney U test depending on distribution. Categorical variables were analysed using chi-square testing. Statistical significance was set at *p* < 0.05.

## Results

### Collective

The data of the patient collective regarding gender and age structure can be found in the table above (see Table 1). There was no significant difference in the structure of the sub-collectives. All patients received a dorsal stabilization with pedicle screw, with or without additive kyphoplasty, cement augmentation and decompression. Cages or similar implants were not used in this collective.

**Table 1:** Overview of the results of the collected parameters.

Patient and procedure characteristics	Total	Navigation	Without navigation	Without navigation 2015–2019	Without navigation 2020–2025	*p*-value
	557	119	438	184	245	
Male	54%	61%	53%	57%	50%	>0,05
Female	46%	39%	47%	43%	50%	>0,05
Age [years]	60,7	65,9	60,7	52,0	64,5	>0,05
Operation time [min]	119	171	105	91	115	<0,05
Screws per surgery	6	8	5	4	6	<0,01
Time per screw [min]	21,6	22,5	21,4	21,6	21,1	>0,05
Cement	26,6%	19,3%	28,5%	12,0%	40,6%	
3D-scan	86,0%	100,0%	82,2%	70,7%	90,6%	
Kyphoplasty	13,3%	6,7%	15,1%	12,5%	16,9%	
Navigation	21,4%	100,0%	0,0%	0,0%	0,0%	
Dosis [cGy/cm^2^]	3,197	3,256	3,181	3,155	3,200	>0,05
Radiation time [min]	6,32	5,07	6,65	6,32	7,07	<0,01
Open	6,1%	10,9%	4,3%	4,9%	3,9%	
Percutaneous	93,9%	89,1%	95,7%	95,1%	96,1%	

### Use of navigation

**Figure 1:**
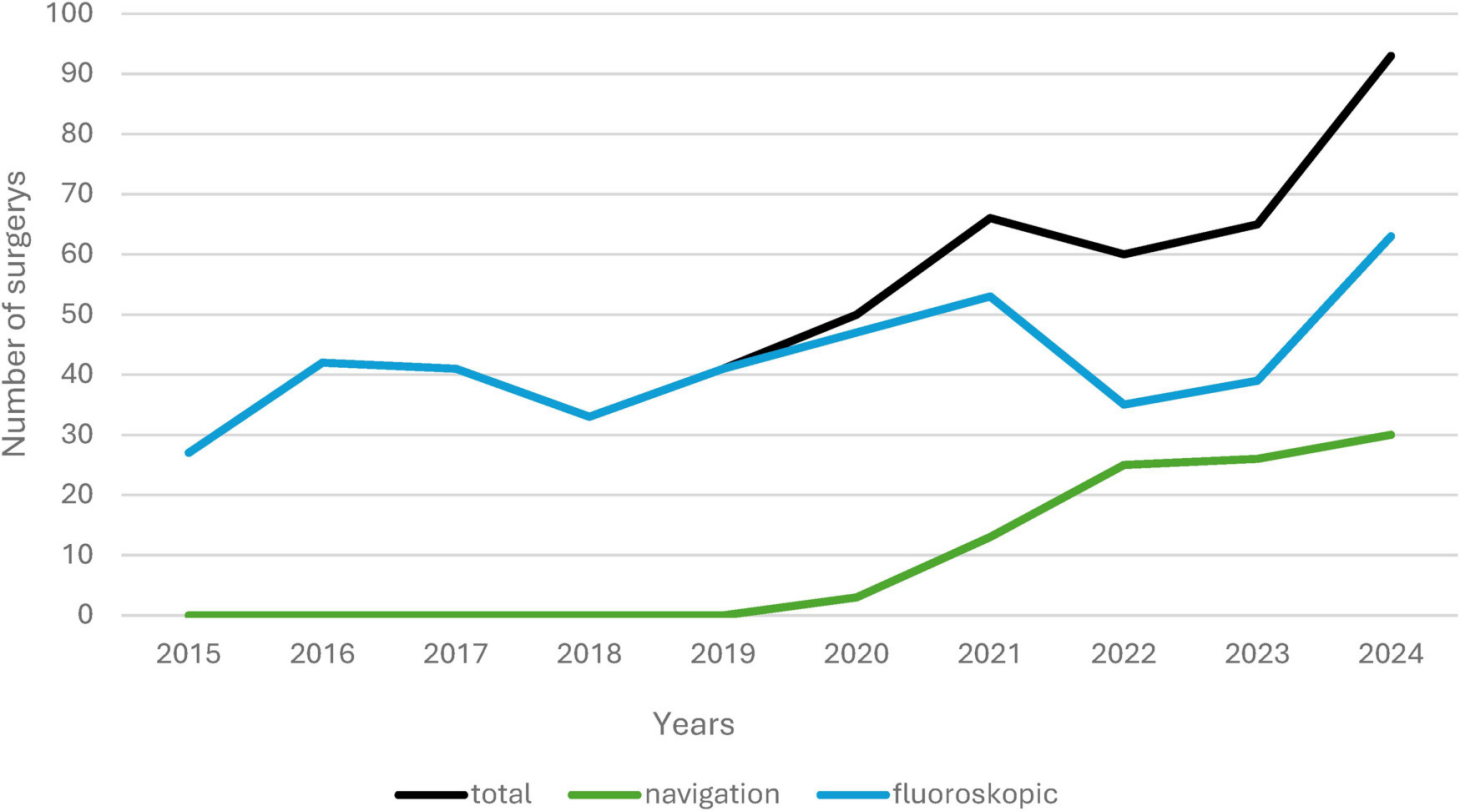
Fluoroscopic and navigated procedures per year.

As can be seen in Figure 1, there has been a steady increase in the number of navigations over the years of use. The year 2025 was only recorded up to and including August, so that the number here is currently even lower than in the previous year. No case required intraoperative conversion from navigation to conventional fluoroscopy due to technical failure or workflow limitations.

### Dose

There was no significant difference in the total dose between navigated and fluoroscopic instrumentation [fluoroscopy 3,181 (33; 54,335; ±3,308) cGy/cm^2^, navigates 3,256 (127; 10,340; ±1,834) cGy/cm^2^, *p* > 0.05]. The radiation time was significantly lower in the navigated procedures than in the fluoroscopic procedures [fluoroscopic 6.6 (0.1; 30:7; ±4.0) min, navigated 5.07 (1.3; 27.0; ±3.4) min; *p* < 0.05].

### Surgery time

The average surgical time of the navigated procedures was significantly longer compared to the non-navigated procedures [navigated 149 (64; 456; ±84) min; non-navigated 105 (22; 392; ±60) min]. With navigation, however, significantly more screws were placed per operation (navigated 8 [1; 20; ±3]; non-navigated 4 [1; 18; ±2]; *p* < 0.01). The surgical time per screw showed no difference between navigated and non-navigated procedures [navigated 22 (11; 116; ±10) min; not navigated 21 (3; 214; ±14) min, *p* = 0.21].

Over the period, there was an improvement in the time per screw in the navigated group from 27 (±22) to 19 (±7) minutes, this improvement was significant (*p* = 0.03). Cement augmentation, kyphoplasty, and adjunctive decompression were each associated with an increase in time per screw of approximately 2–3 min within the respective subgroups, and this difference reached statistical significance. However, when comparing navigation with conventional instrumentation within these subgroups, no statistically significant difference in time per screw was observed.

Overall, 89.8% of all procedures in the period under investigation were operated on within the standard working hours (08:00 to 16:00 average time), 11.2% outside this time.

Overall, 90.2% of the non-navigated procedures in the period under investigation were operated on within the standard working hours (08:00 to 16:00 average time), 9.8% outside this time.

Regarding the percentage of non-navigated interventions before and after the introduction of navigation, no significant difference could be shown here (91.8% before the introduction of navigation, 89% after the introduction of navigation, *p* > 0.05).

A total of 88.2% of the navigated procedures were operated on within the standard working hours (08:00 to 16:00 cutting time), 11.8% outside this time. While at the beginning of the introduction of navigation 100% of the interventions were still operated in the standard working hours, with increasing use a switch was also made for increased use outside the standard working hours. However, this difference was not significant (*p* > 0.05).

### Localization

**Figure 2:**
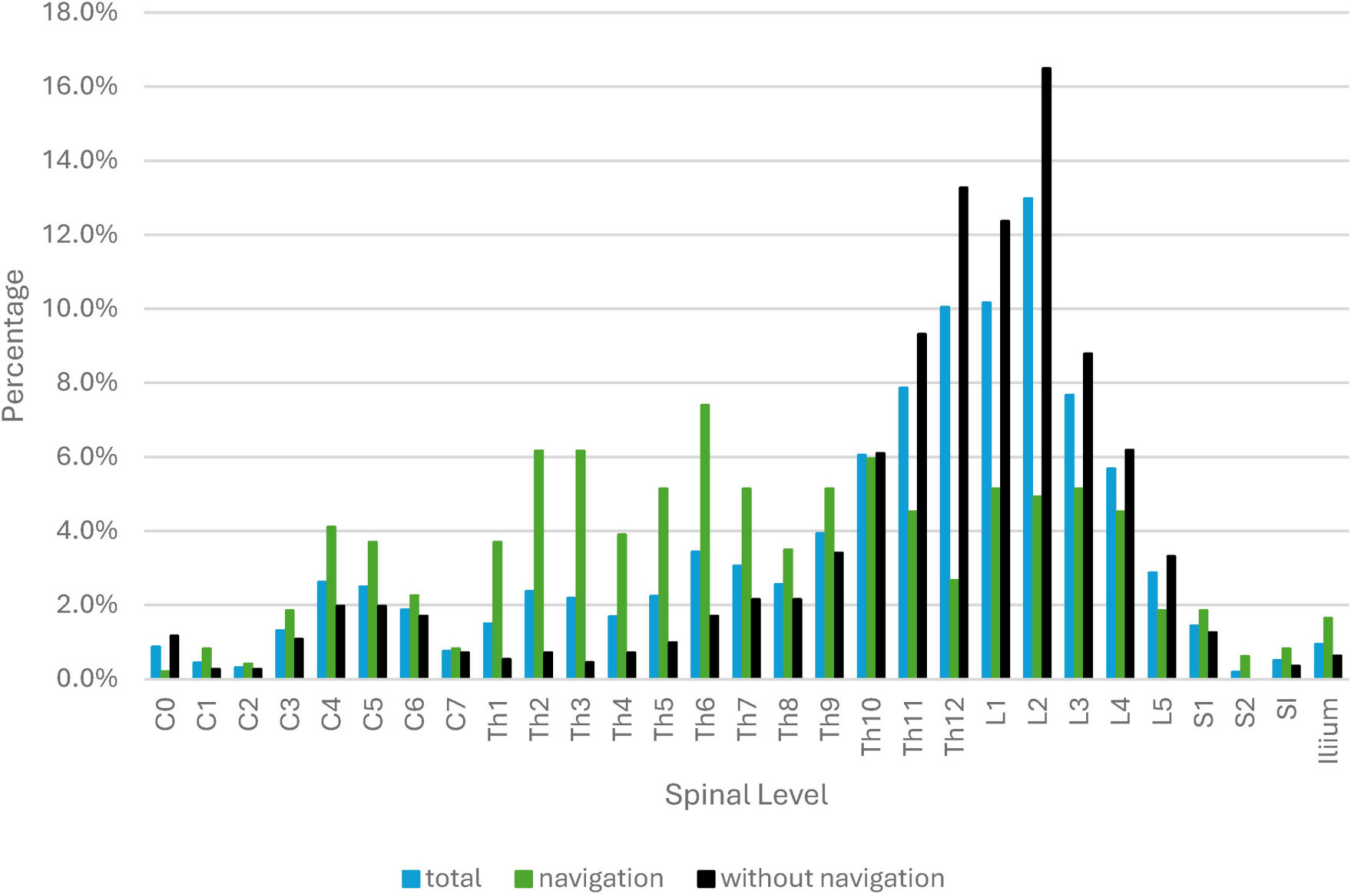
Instrumented vertebral bodies with and without navigation.

Navigation was mainly used around the cervicothoracic junction, the upper to middle thoracic spine as well as the lower lumbar spine and the pelvis/os sacrum. In the lumbar spine area, a percentage of more procedures were performed fluoroscopically (see Figure 2).

In the first years (2020 to 2021), mainly procedures in the lumbar spine area were carried out in a navigated manner. With increasing use, a switch was made here with increasing navigated instrumentation in the upper to middle thoracic spine as well as the lower lumbar spine and the pelvis/os sacrum.

### Revision rates

The revision rate was low in both groups and showed no significant difference (5% navigated, 7% non-navigated, *p* > 0.05). There was a significantly higher revision rate due to screw misalignment in the non-navigated group (1% navigated, 5% not navigated, *p* < 0.05), while in the group of navigated procedures, the number of revisions due to wound healing disorders was increased (navigated 4%, unnavigated 2%, *p* < 0.05).

### ASA classification

**Figure 3:**
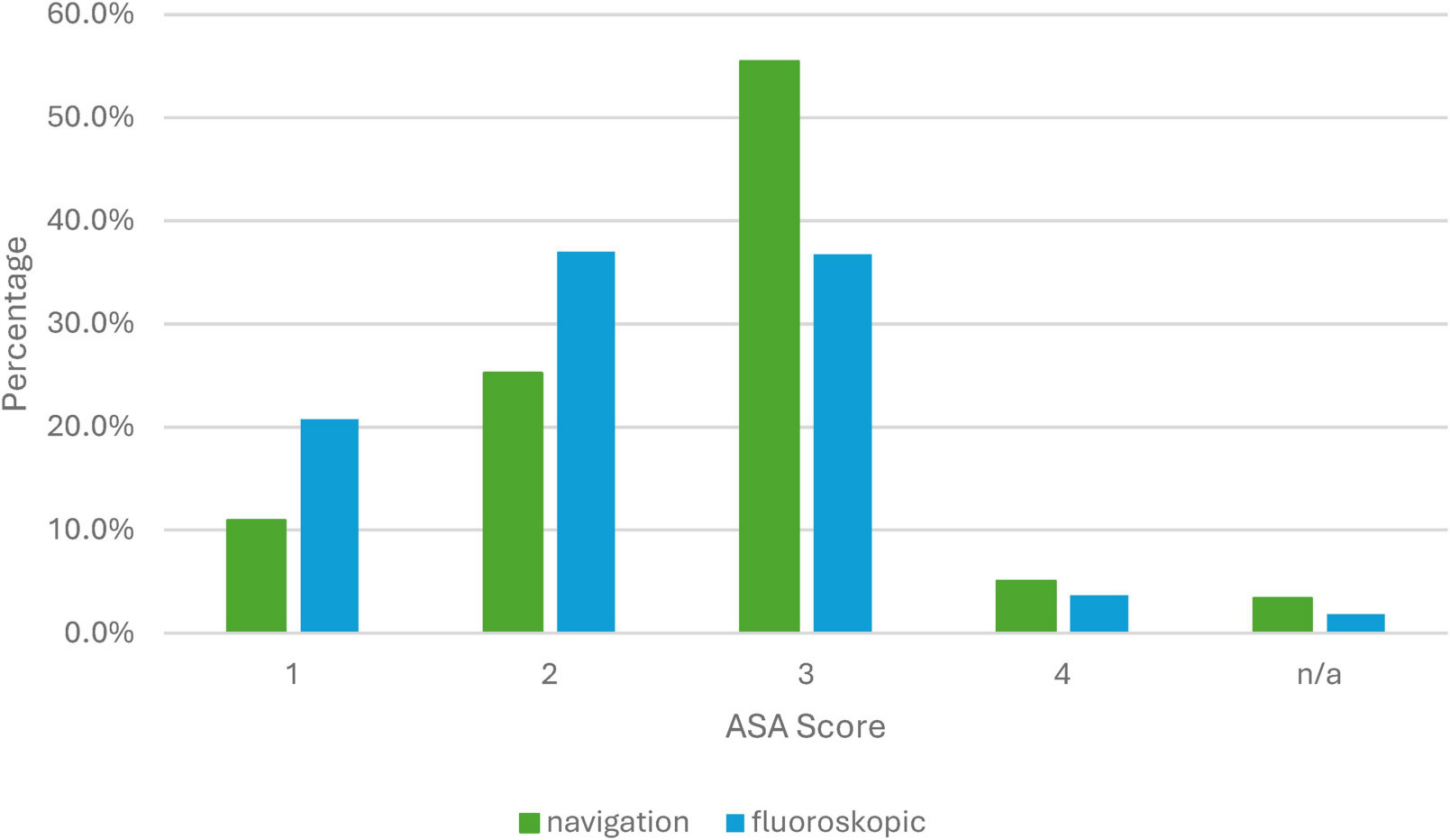
ASA classification.

The distribution of the ASA classification is shown in the figure below (see Figure 3). In the group of navigated procedures, there were significantly more patients with an ASA score of 3 or higher than in the group of non-navigated procedures (*p* < 0,05).

## Discussion

The present study analyzed the utilization of navigation for dorsal spinal instrumentation over a 10-year period at a Level I trauma center and compared procedural characteristics with fluoroscopic techniques. The findings offer insights into changes in surgical workflow, efficiency, and radiation exposure as navigation became increasingly implemented into routine practice. This discussion contextualizes these observations within current evidence and highlights benefits, limitations, and future perspectives of navigated spinal instrumentation.

The steady increase in navigation use from 2020 onwards reflects a broader international trend. Numerous studies have shown that navigation systems have experienced rapid uptake due to accuracy gains and workflow improvements (13).

In our cohort, navigation was initially reserved for lumbar procedures but expanded to more challenging regions such as the cervicothoracic junction, upper thoracic spine, sacrum, and pelvis. This mirrors the literature, where navigation demonstrates its largest benefit in anatomically difficult regions or cases with distorted anatomy (14).

Consistent with existing evidence, navigated instrumentation in this study demonstrated significantly fewer revisions due to screw malposition compared with fluoroscopic procedures. Prior research reports pedicle screw accuracy rates of 95%–98% with navigation, compared to 85%–92% under fluoroscopy**,** depending on anatomical region (15). The low revision rate in our cohort aligns with these findings and supports the notion that navigation is particularly beneficial in regions with narrow pedicles, steep angulation or limited fluoroscopic visibility. Conversely, the higher number of revisions for wound healing complications likely reflects case selection rather than a navigation-related effect, as navigated patients exhibited higher ASA scores and therefore greater systemic vulnerability. This interpretation is consistent with large registry studies identifying comorbidity and prolonged operative time as contributors to postoperative wound complications (16). Also, the higher number of open procedures in die navigated group maybe play a role in the slightly higher number of wound healing problems.

In contrast to degenerative spine surgery, traumatic spinal pathology is characterized by disrupted anatomical landmarks, fracture-related deformity, and intraoperative changes following reduction or positioning. These factors substantially increase the risk of pedicle screw misplacement when relying on fluoroscopy alone. Navigation provides real-time three-dimensional spatial orientation that is largely independent of intact anatomy, thereby offering a distinct advantage in traumatic settings.

Although navigated procedures were associated with longer absolute operative time, this difference was largely attributable to a higher number of screws placed per procedure. When normalized to “time per screw”, no significant difference was found between navigated and fluoroscopic instrumentation. This reflects findings where navigation increases setup and registration time but reduces intraoperative “trial-and-error” screw placement, ultimately balancing overall efficiency (4).

Notably, our data demonstrated a significant reduction of time per screw in the navigated group over time (from 27 to 19 min). This suggests a clear learning curve, which is a well-described phenomenon in navigation-assisted spine surgery. Multiple studies report that familiarity with workflow, improved registration techniques, and integration of navigation into OR logistics lead to rapid efficiency gains after the first cases (17). This trend is an important indicator of system maturation and confirms that navigation becomes increasingly efficient with routine use.

Total radiation dose did not differ significantly between navigated and fluoroscopic procedures in our study. This result is partly influenced by the high rate of 3D scans in both groups (70%–90%), indicating that 3D image acquisition is frequently part of our institutional standard independent of navigation (18). This observation likely reflects institutional workflow characteristics. In our center, intraoperative 3D imaging is frequently performed even in fluoroscopically guided procedures, resulting in a high rate of 3D scans in both groups. Consequently, the additional 3D acquisition required for navigation does not substantially increase the overall radiation dose. Instead, the main difference lies in the workflow of screw placement: navigated procedures require fewer repeated fluoroscopic images during instrumentation, which explains the significantly shorter radiation time.

However, radiation time was significantly lower in navigated procedures. This aligns with the literature: while navigation requires a single 3D dataset at the beginning, conventional fluoroscopy demands repeated imaging during screw placement. Meta-analyses consistently show that navigation reduces surgeon and staff radiation exposure significantly, even when patient dose remains similar due to the initial 3D scan (3).

Patients undergoing navigated procedures in our cohort were older and had significantly higher ASA classifications, suggesting that navigation was preferentially used in more complex cases. Especially in elderly patients with poor bone quality, obesity, pronounced degenerative changes, and complex or distorted spinal anatomy, navigation offers clear advantages, as fluoroscopy in these situations often fails to provide sufficiently reliable anatomical visualization and orientation.

Rather than demonstrating isolated technical advantages, this study illustrates how navigation progressively transformed surgical decision-making, case selection, and workflow integration in a high-volume trauma centre. The observed shift toward anatomically complex regions and high-risk patients represents a pragmatic evolution of clinical practice that is insufficiently addressed in previous reports.

## Limitations

Several limitations must be considered in this analysis. Firstly, this is a retrospective study without blinding, which introduces potential selection bias and limits causal interpretation. Navigated procedures were more frequently performed in older patients with higher ASA classifications, suggesting that navigation was preferentially used in more complex or high-risk cases. These differences may influence operative time, complication rates, and workflow parameters.

Secondly, differences in surgical complexity were not fully controlled, particularly regarding anatomical region and number of screws. Although time per screw was used as a normalized efficiency metric, residual confounding cannot be excluded.

Thirdly, formal radiographic accuracy grading (e.g., Gertzbein–Robbins classification) was not feasible because routine postoperative CT imaging was not performed in all patients. Therefore, revision due to screw misplacement was used as a clinically relevant surrogate marker for screw accuracy.

Finally, the relatively smaller number of navigated procedures limits the statistical power of subgroup analyses.

## Conclusion

The results of this study align closely with the current understanding of navigation-assisted spine surgery, as outlined in the introduction. Consistent with previous reports, navigation was preferentially used in anatomically demanding regions and in patients with higher comorbidity, underscoring its role as a supportive tool in complex or high-risk procedures. The progressive rise in navigated interventions and the shift toward thoracic, cervicothoracic, and pelvic instrumentation demonstrate that navigation systems can actively reshape clinical routines once fully incorporated into institutional workflows.

At our institution, navigation has evolved into an integral component of spinal instrumentation. Although navigated procedures were initially associated with longer operative times, they provided clear advantages in terms of screw accuracy, reduced radiation time, and improved feasibility in anatomically challenging areas. The significant reduction in time per screw over the study period illustrates a pronounced learning curve and emphasizes the importance of surgical experience, team coordination, and workflow optimization. Given the rising complexity of spinal surgery and an increasingly elderly and comorbid patient population, the clinical relevance of navigation is expected to further increase, particularly in demanding anatomical regions. At our Level I trauma center, navigation is firmly established not only during peak operating hours but also outside prime time, reflecting its integration into routine surgical decision-making. Even at times with reduced staffing or limited resources, navigated procedures are consistently performed and currently account for approximately of one third of all spinal cases, serving as a benchmark for standardized high-quality care.

Navigation technologies themselves continue to develop rapidly. Emerging innovations including robotic systems, augmented reality applications, markerless surface registration, and AI-based predictive planning are anticipated to streamline intraoperative workflows, shorten setup and registration times, and further enhance accuracy (19, 20).

Future research should therefore emphasize prospective comparative studies, cost-effectiveness analyses, long-term functional outcomes, and the identification of patient subgroups who benefit most from navigated techniques.

Taken together, our findings demonstrate that the introduction of navigation did not merely add another technical option but gradually transformed surgical decision-making, case selection, and overall operative efficiency within our department. With increasing adoption and expanding indications, navigation has become a valuable asset in everyday practice—particularly for anatomically complex instrumentation and for patients at elevated medical risk. As technological advances continue, navigation is likely to further strengthen its role in improving workflow efficiency and clinical outcomes in spinal trauma surgery.

## Data Availability

The raw data supporting the conclusions of this article will be made available by the authors, without undue reservation.
